# Transcriptome reprogramming, epigenetic modifications and alternative splicing orchestrate the tomato root response to the beneficial fungus *Trichoderma harzianum*

**DOI:** 10.1038/s41438-018-0079-1

**Published:** 2019-01-01

**Authors:** Monica De Palma, Maria Salzano, Clizia Villano, Riccardo Aversano, Matteo Lorito, Michelina Ruocco, Teresa Docimo, Anna Lisa Piccinelli, Nunzio D’Agostino, Marina Tucci

**Affiliations:** 1 Institute of Biosciences and BioResources, Research Division Portici, National Research Council, 80055 Portici, Italy; 2 Department of Agricultural Sciences, University of Naples Federico II, 80055 Portici, Italy; 3 Institute for Sustainable Plant Protection, National Research Council, 80055 Portici, Italy; 4 Department of Pharmacy, University of Salerno, 84084 Fisciano, Italy; 5 CREA, Research Centre for Vegetable and Ornamental Crops, 84098 Pontecagnano Faiano, Italy

**Keywords:** Biotic, Plant symbiosis, Plant genetics

## Abstract

Beneficial interactions of rhizosphere microorganisms are widely exploited for plant biofertilization and mitigation of biotic and abiotic constraints. To provide new insights into the onset of the roots–beneficial microorganisms interplay, we characterised the transcriptomes expressed in tomato roots at 24, 48 and 72 h post inoculation with the beneficial fungus *Trichoderma harzianum* T22 and analysed the epigenetic and post-trascriptional regulation mechanisms. We detected 1243 tomato transcripts that were differentially expressed between *Trichoderma*-interacting and control roots and 83 *T. harzianum* transcripts that were differentially expressed between the three experimental time points. Interaction with *Trichoderma* triggered a transcriptional response mainly ascribable to signal recognition and transduction, stress response, transcriptional regulation and transport. In tomato roots, salicylic acid, and not jasmonate, appears to have a prominent role in orchestrating the interplay with this beneficial strain. Differential regulation of many nutrient transporter genes indicated a strong effect on plant nutrition processes, which, together with the possible modifications in root architecture triggered by ethylene/indole-3-acetic acid signalling at 72 h post inoculation may concur to the well-described growth-promotion ability of this strain. Alongside, *T. harzianum*-induced defence priming and stress tolerance may be mediated by the induction of reactive oxygen species, detoxification and defence genes. A deeper insight into gene expression and regulation control provided first evidences for the involvement of cytosine methylation and alternative splicing mechanisms in the plant–*Trichoderma* interaction. A model is proposed that integrates the plant transcriptomic responses in the roots, where interaction between the plant and beneficial rhizosphere microorganisms occurs.

## Symbiosis: the genetics of beneficial tomato root–fungus interaction

The fungus *Trichoderma harzianum* induces differentiated protein production in tomato roots that benefits plant nutrition and survivability. Microorganisms advantageous to plants have long been exploited in agriculture; however, with limited studies into the interface of *Trichoderma* fungus and plants—the roots. Italian researchers, led by the Research Centre for Vegetable and Ornamental Crops' Nunzio D'Agostino and the National Research Council’s Marina Tucci, inoculated tomato plant roots with *T. harzianum* over 72 h, finding over 1200 examples of differential gene expression and post-expression modification that resulted in improved plant growth and immune system regulation. The interaction also induces a root change that likely promotes further interaction with the fungus and increased stress tolerance via promoting antioxidation and defensive activity. The team’s work provides early evidence of the molecular mechanisms behind root–fungus symbiosis and may help to inform crop breeding and fertilisation strategies.

## Introduction

The need for safeguarding both human and environmental health as well as for preserving natural resources requires an effective crop management combined with a substantially reduced application of agrochemicals. To this purpose, beneficial rhizosphere microbes are being increasingly exploited, for example for biofertilization, disease and pest control, alleviation of environmental constraints^1,2^. Plant growth-promoting fungi (PGPF) include many strains of *Trichoderma* spp., which are also able to colonise roots, behaving as opportunistic symbionts. Along with direct biocontrol of soil pathogens, *Trichoderma* spp. can exert stimulation of the plant immune system (induced systemic resistance, ISR), and pre-activation (priming) of molecular mechanisms of defence against a broad range of pathogens^3–7^. In addition, positive effects of these PGPF on plant growth and alleviation of abiotic stresses have been widely reported^8–10^.

The cascade of molecular events that characterises the onset of the plant–*Trichoderma* interaction has been documented by several proteomic and transcriptomic studies, mainly on the aboveground part of the plant^11–14^. Perception of microbe-associated molecular patterns (MAMPs) by pattern recognition receptors (PRRs) mediates early perception and activates MAMPs/DAMPs-triggered immunity (MTI/DTI)^15,16^. To date, only a few genes coding for receptor/recognition protein–*Trichoderma* elicitor pairs have been characterised^17^. *Trichoderma* effectors have also been suggested to activate effector-triggered immunity (ETI)^17^. Indeed, *Trichoderma*-stimulated cell death during root colonisation^18^, a phenomenon that is suggestive of ETI-induced programmed cell death. Among the early events induced by host*-Trichoderma* recognition, both salicylic acid (SA)-mediated and jasmonate (JA)/ethylene (Et)-mediated signalling have been implicated, but also abscisic acid (ABA) and indole-3-acetic acid (IAA) have been proposed to play important roles^17,19^. Recent studies indicate that regulatory mechanisms, such as epigenetic (e.g. DNA methylation, histone modification) and post-transcriptional (e.g. alternative splicing, AS) modifications, are key pathogenesis modulators^20,21^ and could also be involved in establishing beneficial interactions^21^. In potato, higher cytosine DNA methylation is implicated in suppression of the bacterial endophyte *Burkholderia phytofirmans*-induced plant growth stimulation^22^, suggesting that DNA (de)methylation could be relevant to beneficial interactions. Recently, evaluation of AS patterns from different plant species, including tomato, revealed that 39–70% of multi-exon genes produces at least one splice variants^23^ and that most genes related to plant defence undergo AS during plant–pathogen interactions^20,24,25^. However, AS remains poorly studied in plants and even less in Solanaceous species^26,27^.

Despite the fact that recognition of rhizosphere-competent *Trichoderma* strains and the elicitation of plant responses are exclusively mediated by the root system^28–30^, investigations on the belowground interplay between roots and beneficial fungi have so far been limited^8,30,31^. In such plant organ, the few available data indicate that *A. thaliana* colonisation by *T. asperelloides* T203 requires activation of the JA pathway and enhanced expression of specific WRKY transcription factors (TFs), which stimulate JA signalling via suppression of jasmonate ZIM domain (JAZ) repressors^8^. SA was suggested to be involved in limiting *Trichoderma* root colonisation based on results with *T. harzianum* after interaction with the SA-impaired *sid2* mutant of *A. thaliana*^32,33^ or with oil palm roots^31^. *Trichoderma* also activates an efficient reactive oxygen species (ROS) detoxification system and production of antimicrobial compounds through the phenylpropanoid pathway, which may participate in limiting pathogen infection, but also in ameliorating plant tolerance to abiotic stresses^3,8,34,35^. Members of the antioxidant machinery activated by *Trichoderma* spp. include antioxidant enzymes, such as superoxide dismutase (SOD), peroxidases (PODs) and gluthatione S-transferases (GSTs) in roots^8,30,35^ and GSTs or glutaredoxin (GRX)/thioredoxin (TRX) in plants^12,13^.

In the present study, we report results of the whole-transcriptome analysis of *Solanum lycopersicum* roots inoculated with *T. harzianum* T22, a PGPF widely used in several biofertilizer and biopesticide formulations^36,37^. The focus on this organ allowed us to provide a root-specific model integrating the main signalling events and regulation mechanisms occurring during early root colonisation by *Trichoderma*, at time points (24–72 hpi) that were chosen on the basis of previous findings, to coincide with relevant stages of fungal colonisation^29^. Moreover, we identify, for the first time, the gene *loci* putatively affected by AS during the interaction with *Trichoderma* as well as provide evidence for a considerable number of previously unannotated AS events in tomato roots.

## Results

### RNA sequencing and read mapping onto *S. lycopersicum* and *T. harzianum* reference genomes

We carried out a NGS-based global transcriptomic analysis of *S. lycopersicum* roots interacting with a beneficial fungus of the rhizosphere. RNA-seq of *T. harzianum* T22-treated (T) and untreated (C) tomato roots at three time points (24, 48 and 72 hpi) generated a number of raw reads ranging from 46,114,872 (C72) to 57,069,524 (C24; Table 1). Approximately 13.5% of the reads were filtered out based on sequence quality, while a further ~5% were discarded as a consequence of trimming operations. The percentage of high-quality reads mapped onto the tomato reference genome was >93% for all samples (Table 1). The subset including all unmapped reads was aligned onto the *T. harzianum* genome. Numbers and percentages of total and uniquely mapped reads onto the fungus genome are shown in Table 1. As expected, very few reads from C samples were successfully aligned. By contrast, a considerable number of uniquely mapped reads from T samples were identified, which increased with the time of interaction, together with the amount of the fungus in the tomato roots, as estimated by qRT-PCR of *T. harzianum* actin (Fig. S1). Box plots describing the distribution of read counts before and after normalisation are shown in Fig. S2. High Pearson’s correlations between sequencing replicates were found, with average *r*^2^ values ranging from 0.96 to 0.99 (Fig. S3).

**Table 1:** Sequencing and alignment statistics of the control-inoculated (C) and *T. harzianum* T22-inoculated (T) tomato root samples at 24, 48 and 72 h of interaction

Sample	Raw reads (no.)	High-quality reads (no.)	Reads aligned onto the *S. lycopersicum* genome (no.)		Reads aligned onto the *T. harzianum* genome (no.)	
			Total mapped	Uniquely mapped	Total mapped	Uniquely mapped
C24	57,069,524	46,879,826	44,354,968 (94.6%)	43,190,733 (97.4%)	3 (–)	–
C48	49,480,290	40,703,519	38,059,461 (93.5%)	37,101,879 (97.5%)	10 (–)	–
C72	46,114,872	37,277,990	35,376,200 (94.9%)	38,442,807 (98.2%)	7 (–)	–
T24	47,457,859	38,753,059	36,681,531 (94.7%)	35,789,320 (97.6%)	11,309 (0.54%)	11,299 (~100%)
T48	50,908,596	41,928,916	39,703,689 (94.7%)	38,720,180 (97.5%)	24,014 (1.05%)	23,974 (~100%)
T72	52,392,405	43,442,331	41,085,823 (94.6%)	39,996,180 (97.3%)	32,288 (1.40%)	32,238 (~100%)

For each sample, percentages of uniquely mapped reads (onto tomato or fungus genomes) were calculated based on the corresponding 'Total mapped' values. For the alignment onto the *T. harzianum* genome, the subset including all unmapped reads onto the tomato genome was used as input data. The given values are the average of three biological replicates

### *S. lycopersicum* genes differentially expressed in T22-treated plants: a specific plant response already after 24 h

By comparing T vs. C samples, 1243 tomato DEGs were identified over the three time points. Only the DEGs detected by both EdgeR and DESeq were considered (Table S2). RNA-seq data on 12 genes, randomly selected between upregulated and downregulated DEGs, were validated by real time (RT)-qPCR and demonstrated good agreement between the two methods of gene expression profiling (mean Pearson’s correlations coefficient: 0.85), with the exception of two genes (Solyc06g073530 and Solyc10g083230; Fig. S4).

**Fig. 1:**
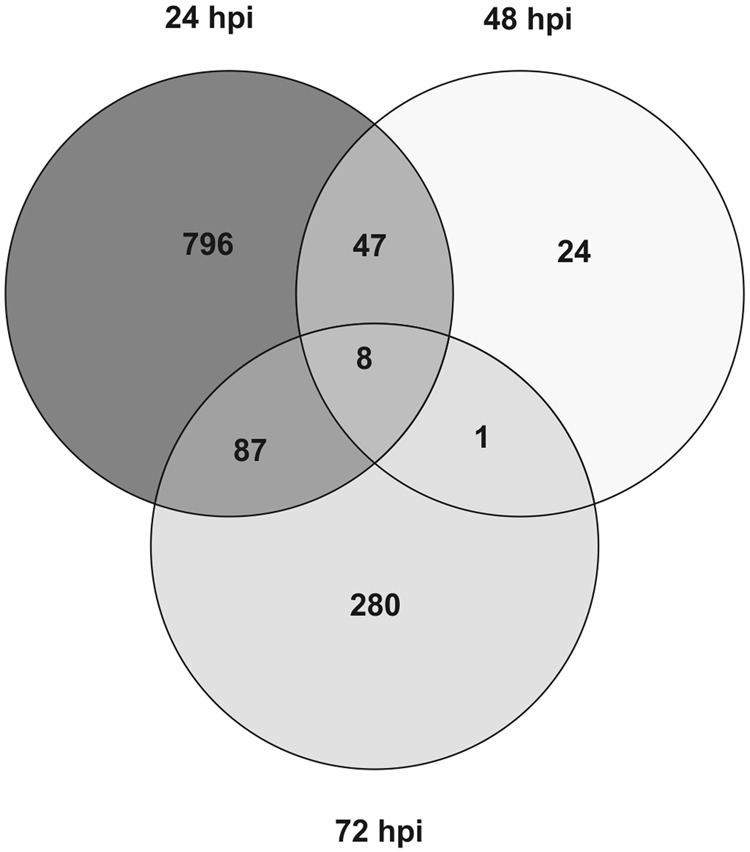
Venn diagram of tomato's differentially expressed genes in *T. harzianum* T22-treated vs. control roots. The distribution of the 1243 differentially expressed genes (DEGs) in tomato root resulting from RNA-seq analysis is reported for different times of interaction (24, 48 and 72 hpi) with the beneficial fungus *T. harzianum*. DEGs were identified setting the false discovery rate at 10% (*p* < 0.1) and the minimum fold change at ±1.1

The highest number of DEGs (938) was found at 24 h of interaction with *T. harzianum* (Fig. 1), the lowest (80) at 48 hpi. They were mainly downregulated at 24 and 72 hpi (531 vs. 407 and 222 vs. 154, respectively), while the opposite was true at 48 hpi (35 vs. 45) (Fig. 1 and Table S2). A gene with unknown function (Solyc01g090980) was the most induced at both 24 (log_2_ FC: 8.23) and 48 hpi (log_2_ FC: 6.36). Sequence analysis revealed that this gene codes for a protein with the domain DUF4535 (InterPro ID: 027854), suggesting that it may be a short secreted protein. On the other hand, the most downregulated gene (Solyc10g075150, log_2_ FC: −5.33) was identified at 72 hpi and codes for a non-specific lipid-transfer protein (ns-LTP), which was not differentially expressed at 24 and 48 hpi.

As far as *Trichoderma* transcripts, the complete list of DEGs and their distribution across the three time points are shown in Table S3 and Fig. S5.

**Fig. 2:**
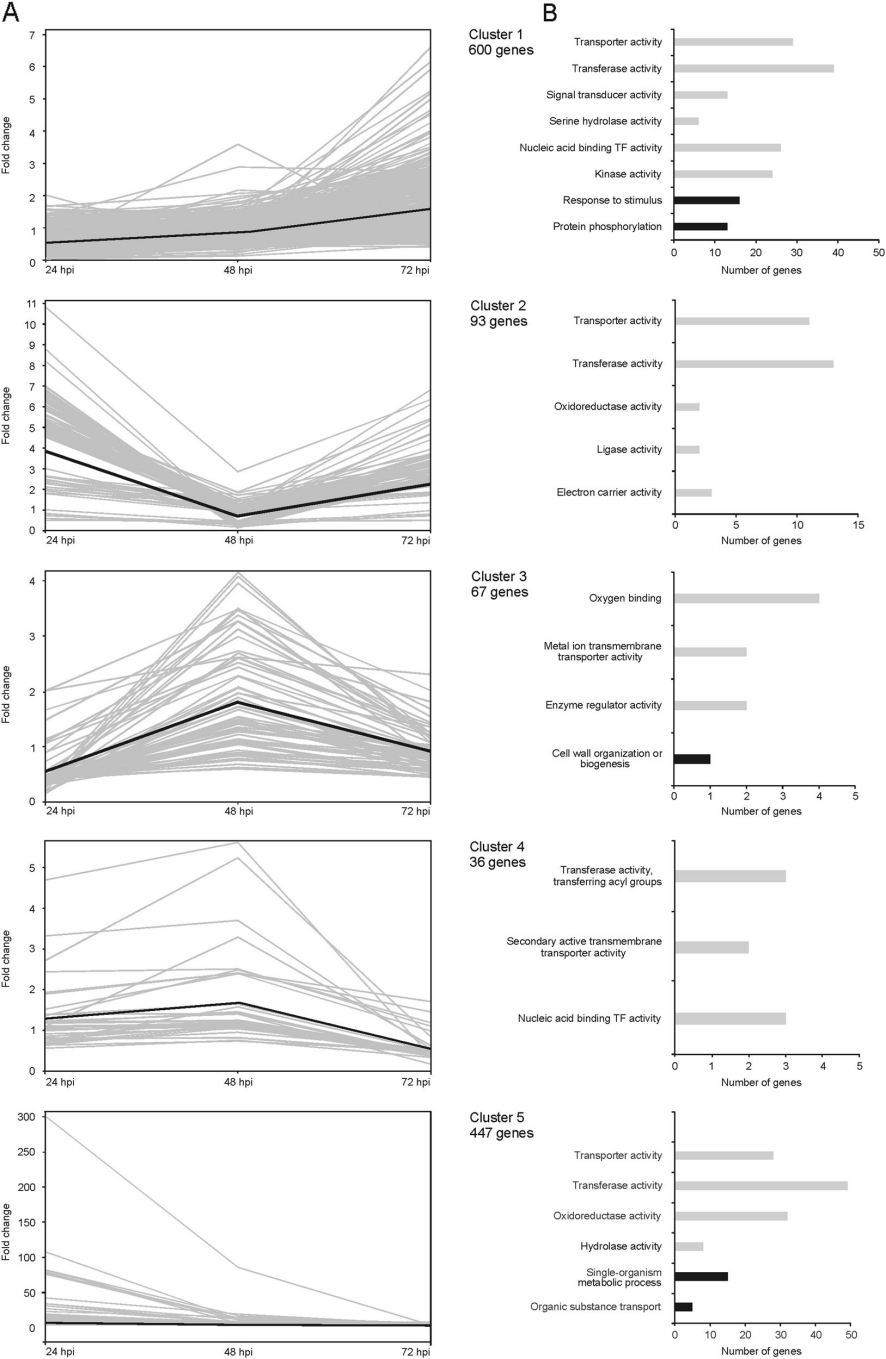
Clustering of expression profiles of tomato root genes differentially expressed during the interaction with *T. harzianum* T22 and gene ontology enrichment analysis. **a** Grouping of tomato genes modulated by *T. harzianum* into five clusters, according to their expression profiles across the interaction period (24, 48 and 72 hpi), using the Pearson’s correlation distance (SOTA method). The number of genes assigned to each cluster is indicated. The thick line indicates the cluster centroid. The *y*-axis represents the fold change of the gene expression level. **b** Significantly enriched gene ontology (GO) terms associated with each expression cluster, ordered according to increasing *p*-value. Black and grey bars represent the biological process (BP) and molecular function (MF) categories, respectively. The *x*-axis represents the number of genes grouped in each GO category. TF, transcription factor

According to similarities of expression trends across the three time points, the SOTA analysis grouped the 1243 tomato DEGs in five clusters (Fig. 2a, Table S2). GO analysis highlighted that in most SOTA clusters, the largest molecular function (MF) categories affected by *Trichoderma* treatment were ‘transferase activity’, followed by ‘transporter’ and ‘transmembrane transporter’ activities (Fig. 2b, Table S4). ‘Nucleic acid binding’ (transcription factor) activity is one of the largest MF categories represented in cluster 1, which groups genes that showed downregulation within the first 48 hpi and a weak upregulation at 72 hpi. Cluster 1 includes also many genes coding for proteins with ‘kinase’ and ‘signal transducer’ activities that are possibly involved in immune response; indeed, the biological process (BP) category ‘response to *stimulus*’ is overrepresented in cluster 1 (Fig. 2b, Table S4). Furthermore, cluster 1 comprises genes classified in the ‘serine hydrolase activity’ MF that, similarly to the ‘hydrolase activity’ genes in cluster 5, which groups genes upregulated at 24 hpi, not differentially expressed at 48 hpi and weakly downregulated at 72 hpi, are related to protein modification. Genes having ‘oxidoreductase activity’ were strongly represented in cluster 5, which groups genes upregulated at 24 hpi, not differentially expressed at 48 hpi and weakly downregulated at 72 hpi (Fig. 2b, Table S4).

### Tomato interaction with *Trichoderma* triggers transcriptional reprogramming of genes involved in defence, metabolism and transport

**Fig. 3:**
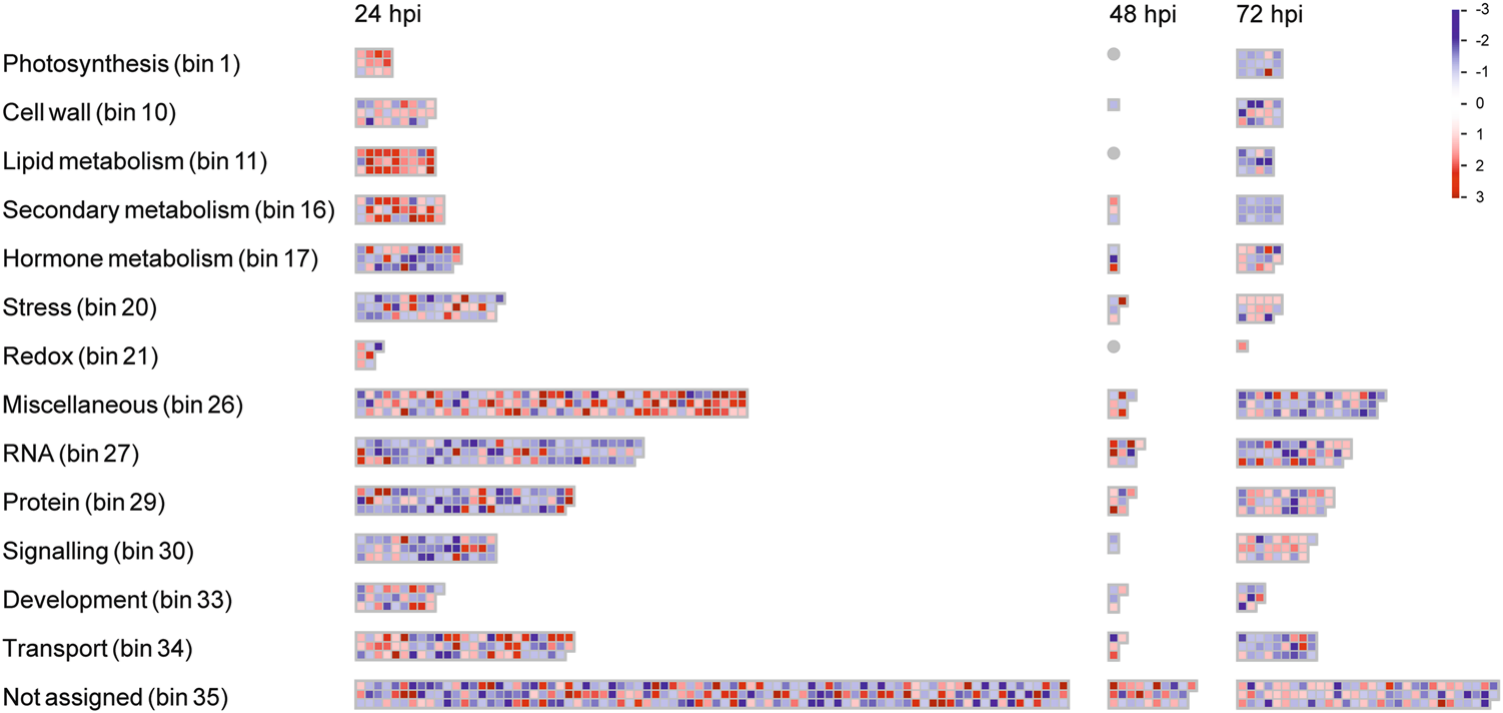
MapMan analysis of tomato root genes differentially expressed during the interaction with *T. harzianum* T22. Organisation of tomato root's differentially expressed genes (DEGs) in functional categories (bins) according to the MapMan ontology across the interaction period (24, 48 and 72 hpi). Genes significantly upregulated and downregulated in treated vs. control plants are indicated in red and blue, respectively. The colour set scale is on top right corner

DEGs distribution according to the MapMan ontology confirmed the modulation of tomato plant response to external *stimuli*, as highlighted by the over-representation of bins related to recognition, signalling and general stress response across the three time points (Fig. 3).

‘Signalling’ (bin 30) included DEGs mainly downregulated at 24 and 48 hpi and upregulated at 72 hpi. DEGs associated with the Et, SA and auxin pathways were found in ‘Hormone metabolism’ (bin 17). Most DEGs within this bin were inhibited at 24 and 48 hpi and slightly induced at 72 hpi.

DEGs associated with general response to *stimulus* were classified in several bins. In particular, increased expression at 24 hpi followed by downregulation at 72 hpi was shown by most genes coding for cell wall-modifying enzymes (bin 10), by key genes of the phenylpropanoid pathway (bin 16) and by genes of the antioxidant enzyme machinery (bin 26). Interestingly, antioxidant and redox-sensing mechanisms were also triggered at 24 hpi in response to *Trichoderma* (bin 21). On the contrary, biotic and abiotic response-associated DEGs (bin 20) showed a general repression at 24 hpi, followed by a slight induction at later time points.

At all time points, a large number of DEGs (~26%, ~35% and ~23% at 24, 48 and 72 hpi, respectively) were mapped to the category of ‘Not assigned’ genes (bin 35), including several genes related to general defence response. Within bin 35, 155 DEGs annotated as unknown protein in iTAG were further investigated by constructing a neighbour-joining phylogenetic tree (Fig. S6). This analysis highlighted a cluster of nine sequences with a conserved gamma-thionin motif that characterise defensin proteins. Most genes coding for these putative defensins were downregulated at 24 hpi.

Transcriptomic analysis also evidenced extensive modulation of genes involved in metabolic processes during the early phases of *Trichoderma* root colonisation. ‘Photosynthesis’ (bin 1) included DEGs related to Calvin cycle or coding for structural proteins with a specific role in the photosynthetic machinery that were mainly upregulated at 24 hpi and repressed at 72 hpi (~87% of down-regulated genes). ‘Lipid metabolism’ (bin 11) counted DEGs with roles in fatty acid and phospholipid synthesis or elongation, or coding for lipid transfer proteins (LTPs). In addition, ‘Protein’ (bin 29) included DEGs mostly related to post-translation modification (e.g. kinase) and protein degradation (protease/peptidase) at 24 and 72 hpi.

Several DEGs were included in ‘Transport’ (bin 34) over the experimental time course, being mostly upregulated within the first 48 h. More in detail, we observed modulated expression of nutrient transport (monomers, ions and metals) and ATP-binding cassette (ABC) transporter genes.

The above responses were supported by a large recruitment of the transcriptional machinery (bin 27; Fig. 3), with 139 differentially expressed TFs over the three time points. At 24 hpi, 82% of the DEGs were repressed, while at 72 hpi an even distribution between upregulated and downregulated genes was observed. Numerous MYB (24), ERF (15) and WRKY (13) genes were identified, although other TF classes (bHLH and zinc finger family factors) were also represented.

### *Trichoderma* induces modifications in tomato transcriptional and post-transcriptional regulation

**Fig. 4:**
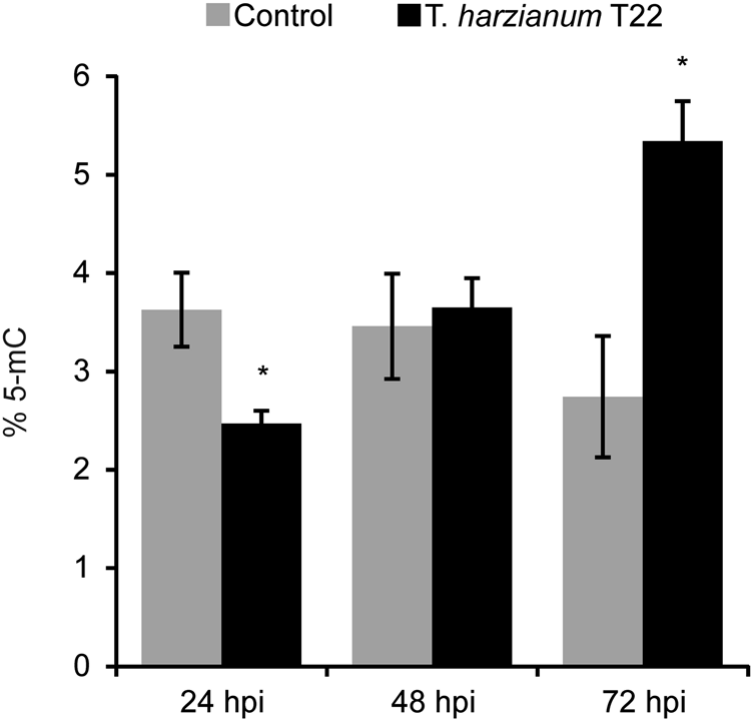
Absolute levels of global DNA methylation in control and *Trichoderma*-treated tomato roots. DNA methylation was assessed across the interaction period (24, 48 and 72 hpi) and reported as percent content of 5-methylCytosine (% 5-mC) using an antibody-based colorimetric detection kit. Methylation levels significantly different from the corresponding control are indicated by *p < 0.05

To provide further insights into gene expression and regulatory control in the plant root–*Trichoderma* interaction, we analysed epigenetic, namely DNA methylation, and post-transcriptional, namely AS, modifications. Interrogation of the *Trichoderma*-induced tomato root DEGs for epiregulators highlighted the modulation of five histone acetyltransferases (HATs) of the GNAT superfamily (known as HAG: histone acetyltransferase GCN5) and of components of the RNA-directed DNA methylation complex (Table 2). Transcription of *SIHAG17* (Solyc08g068280) was activated after 24 hpi and repressed at 72 hpi. *SIHAG8*, *SIHAG15* and *SIHAG*18 (Solyc03g116860, Solyc00g272810 and Solyc12g096840) were suppressed at a very early stage of interaction and resulted as not active thereafter; by contrast, *SIHAG*20 (Solyc08g068710) and *SIJMJ5* (Solyc08g076390, similar to histone demethylase (HDM) of the Jumonji family) were upregulated not earlier than 72 hpi. We also found that the genes coding for RNA-directed DNA methylation 1 (*SIRdM1*, Solyc09g082480), a component of the RNA-directed DNA methylation effector complex, and for Argonaute (AGO) slicer protein (*SIAGO4-like*, Solyc06g073530) were differentially expressed. In particular, the former gradually decreased its expression across the three time points (log_2_ FC: 6.3, 3.7 and 1.8, respectively), the latter was highly suppressed at 24 hpi and finally evened the control expression level (log_2_ FC: −5.2, −2.9, not differentially expressed). To verify whether the gene expression differences found for the RdDM players were also accompanied by cytosine methylation changes, we measured the global DNA methylation levels (in terms of percent content of 5-methylCytosine (% 5-mC)) in the C and T samples at each time point (Fig. 4). Our results showed that control plants have no differences in methylation levels at the three time points; on the contrary in treated roots, we found a decrease in DNA methylation at 24 hpi and an increase at 72 hpi (*p* < 0.05). No statistically significant differences were found in treated roots at 48 hpi post treatment.

**Table 2:** Transcript abundance, expressed in log_2_ FC, of five histone acetyltransferases GCN5 (*SlHAG8, SlHAG15, SlHAG17, SlHAG18 and SlHAG20*), a histone demethylase (*HDM*) of the Jumonji family (*SlJMJ5*), a RNA-directed DNA methylation 1 gene (*SlRdM1*) and a Argonaute slicer (*SlAGO4*-like) observed in tomato roots after 24, 48 and 72 h of interaction with *T. harzianum* T22

Gene abbreviation	iTAG 2.4 ID	Log_2_ FC		
		24 hpi	48 hpi	72 hpi
*SlHAG8*	Solyc03g116860	−1.69	*−0.39*	*−0.13*
*SlHAG15*	Solyc00g272810	−1.25	*−0.93*	*−1.02*
*SlHAG17*	Solyc08g068280	2.37	*−0.14*	−1.63
*SlHAG18*	Solyc12g096840	−2.10	*−0.72*	*0.92*
*SlHAG20*	Solyc08g068710	*0.40*	*0.54*	1.25
*SlJMJ5*	Solyc08g076390	*0.14*	*0.34*	1.23
*SlRDM1*	Solyc09g082480	6.30	3.75	1.84
*SlAGO4-LIKE*	Solyc06g073530	−5.24	−2.99	*−1.17*

For each gene, the iTAG (version 2.4) ID is reported. FC values below the ±1.1 threshold or not satisfying the FDR cutoffs are shown in italic

**Fig. 5:**
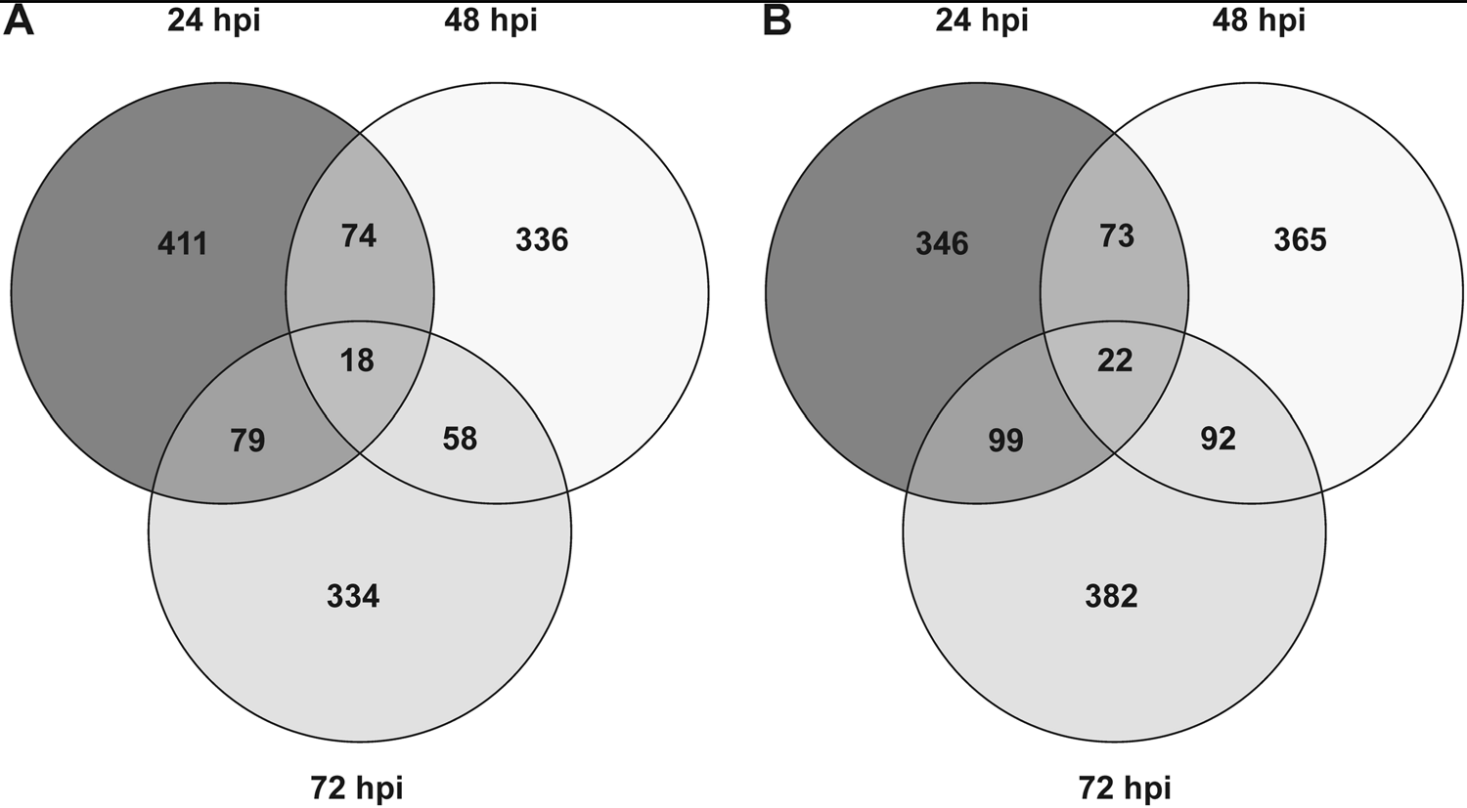
Venn diagrams of tomato gene *loci* affected by alternative splicing in control and *Trichoderma*-treated roots. Distribution of novel transcribed isoforms and/or gene features (‘intron only’, ‘multiple annotation’ and ‘internal exon extension’ categories) resulting from comparison with iTAG2.4 annotations for **a** control and **b** *Trichoderma*-treated root samples at 24, 48 and 72 hpi. R-SAP analysis was run with IdentityCutoff = 95 and CovCutoff = 98

The role of post-transcriptional gene regulation through AS in tomato response to *Trichoderma* was assessed using R-SAP analysis. No differences between C and T samples at each time point were observed in terms of reads belonging to different R-SAP categories. On an average, 31% of the reads are indicative of putative alternative transcriptional events (R-SAP categories: ‘intron only’, ‘multiple annotation’ and ‘internal exon extension’). While the number of genes affected by splicing events was similar in all samples (Fig. 5a, b), the list of genes varied between C and T samples at the three time points. GO-enrichment analysis of genes affected by AS and specific for C or T samples revealed significant over-representation at 24 hpi of the BP term 'response to stimulus', in both *T. harzianum*-treated and control roots. Enriched categories found only in T samples were 'regulation of metabolic process' (at 24 hpi, with most genes annotated as TFs), 'carboxylesterase activity' (at 48 hpi, including gene involved in lipid metabolism) and 'oxigen binding' (at 48 and 72 hpi, with almost all genes annotated as cytochrome P450, involved in secondary metabolism) (Table S5).

## Discussion

**Fig. 6:**
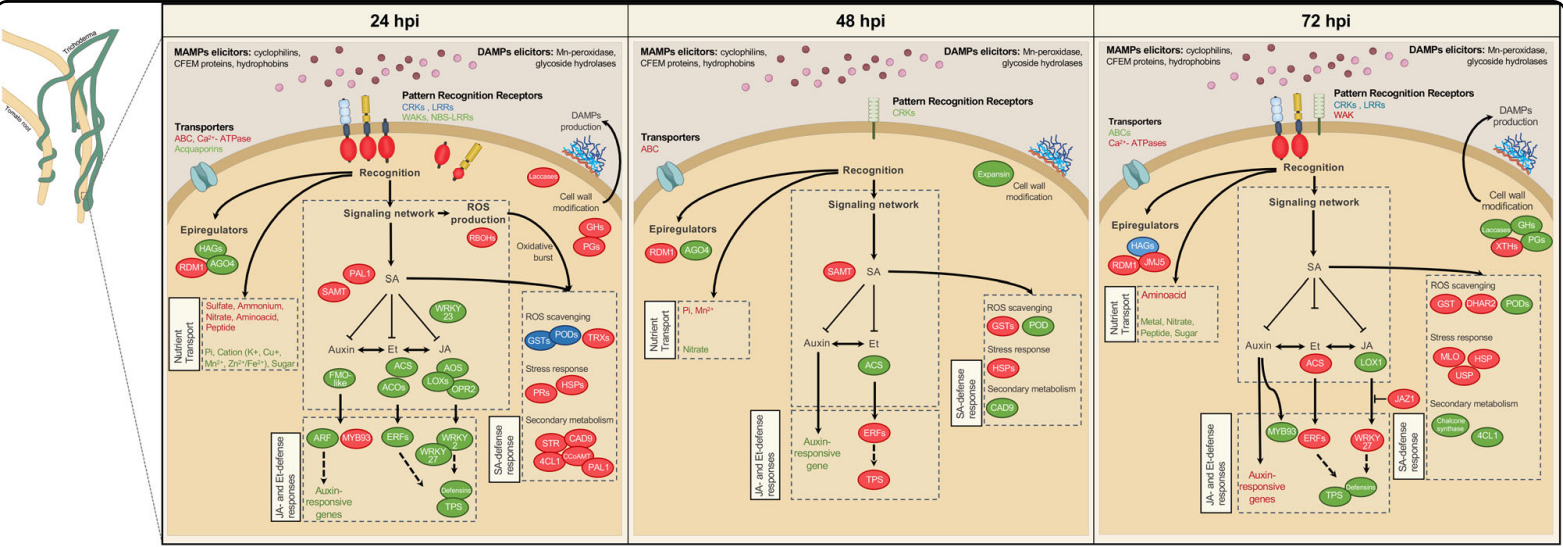
Overview of modulated gene expression in tomato roots during *T. harzianum* T22 colonisation. The illustration is based on annotation of the DEGs in iTAG2.4 and assignment to specific functional categories by MapMan ontology. Tomato genes that were significantly upregulated and downregulated in response to *T. harzianum* at each time point (24, 48 and 72 hpi) are in red and green, respectively. Gene families including both downregulated and upregulated members are in blue. *Trichoderma* transcripts involved in putative MAMP/DAMPs elicitation are also indicated. Solid and dashed lines represent established and hypothesised activities, respectively

Plant root is the crucial organ for perception and recognition of soil microorganisms as well as for the transduction of response signals. However, whole-transcriptome studies of the interplay between roots and beneficial strains of the rhizosphere fungus *Trichoderma* have so far been limited^8,31^. In this study, we detected large transcriptomic perturbations of *T. harzianum*-treated tomato roots across an experimental period ranging from 24 to 72 hpi. Alongside, the increasing amount of fungus and number of *Trichoderma*-specific reads within the roots suggests successful colonisation. Overall, our findings highlight modulation of tomato root gene expression that supports fungal growth and mediates the onset of a positive interaction with these beneficial fungi, eventually resulting in improved plant growth and tolerance to biotic and abiotic stresses. These modifications are discussed below and summarised in Fig. 6.

### Recognition between tomato and *Trichoderma* activates ROS signalling, SA responses and cell wall modifications already at 24 hpi

At 24 hpi, most of the molecular signals related to the interaction between roots and *Trichoderma* have taken place. Putative MAMPs/DAMPs elicitors, identified in our trascriptome as produced by *T. harzianum* T22 (including cyclophilins, CFEM domain proteins, hydrophobins, Mn-POD and glycoside hydrolases) or derived from structural modifications of the plant cell wall (polygalacturonases, PGs; xyloglucan endo-transglucosylase/hydrolase, XTHs; glycoside hydrolases, GH; laccases) (Fig. 6), were apparently recognised by root receptors, as indicated by persistent upregulation of genes encoding membrane-localised LRR proteins, cysteine-rich receptor kinases (CRKs) and by activation of a wall-associated kinase (WAK) at 72 hpi. As a hallmark of successful recognition, we observed increased transcription of tomato respiratory burst oxidase homologues (RBOHs; Fig. 6), which impinges on ROS production^38,39^. Expectedly, ROS accumulation drove a strong induction of the tomato root antioxidant machinery, confirmed by the activation of several related genes, such as GSTs, TRXs and PODs. These findings are consistent with the data by Salas-Marina et al.^30^ and Brotman et al.^8^ in *Arabidopsis* after early colonisation by *T. atroviride* or *T. asperelloides*, respectively. Enhanced expression of genes coding for ABC membrane transporters at 24 and 48 hpi and for components of calcium-mediated or calmodulin-mediated signalling at 24 and 72 hpi (Fig. 6) may concur in the root–Trichoderma cross-talk^16,40^.

**Fig. 7:**
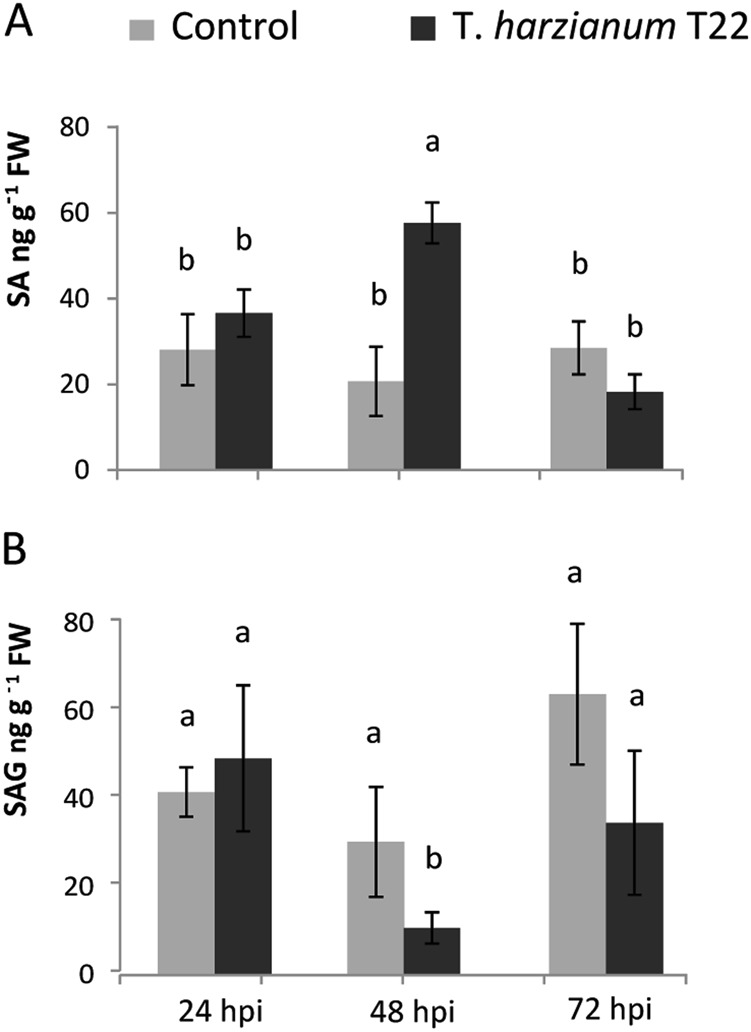
Levels of salicylic acid (SA) and salicylic acid glucoside (SAG) in tomato roots. The levels of **a** SA and **b** SAG were determined in control and *T. harzianum*-treated roots across the interaction period (24, 48 and 72 hpi) by quantitative HPLC-MS/MS. Bars represent the means ± SD of three biological replicates. Data were subjected to analysis of variance and tested for significance (*p* < 0.05) using the Tukey’s test. FW, fresh weight

Several studies have indicated the importance of SA, JA and Et regulation for the response of the aerial part of the plant to *Trichoderma*^4,13,41^. However, the role of specific hormones has not been clarified yet, and seems to be dependent on the experimental conditions and organisms involved^17^. Moreover, their role in the roots has been addressed by fewer studies^8,31^. In our experimental system, increased expression of SA biosynthesis and modification (phenylalanine ammonia lyase, *Pal1*-like and SA-dependent carboxyl methyltransferase, SAMT) and SA-responsive (e.g. pathogenesis-related, PR genes) genes suggests SA accumulation. This in turn may be responsible for the observed transcriptional repression of JA biosynthesis (allele oxide synthase, AOS; lipoxygenases, *Lox1* and *Lox5*, oxo-phytodienoic acid reductase 2, *OPR2*) and JA-responsive genes (e.g. WRKY2, defensins) across the experimental time points (Fig. 6). In addition, differential expression of *SlWRKY2*, *SlWRKY23* and *SlWRKY27*, with sequence similarity with *A. thaliana* TFs (*AtWRKY40*, *AtWRKY22* and *AtWRKY41*) implicated in JA-signalling and SA/JA cross-talk^8,42,43^, further indicates negative regulation of JA-mediated responses. In line with the enhanced expression of SA-related genes, we observed early SA increase in *Trichoderma*-treated tomato roots, with a significant almost threefold accumulation over the respective control at 48 hpi (57.6 vs*.* 20.7 ng g^−1^ FW; Fig. 7a), in contrast with no SA accumulation reported for cucumber roots inoculated with *Trichodema asperellum*^44^. The detected increase in SA was paralleled by a similar decrease of salicylic acid glucoside (SAG), mainly at 48 hpi (10.7 vs. 30.7 ng g^−1^ FW; Fig. 7b), consistently with the suggested role of SAG as an inactive storage form, which needs to be converted to SA to induce SA-related defences^45^. The temporary induction of SA confirms a possible role in avoiding excessive *Trichoderma* penetration within the roots^32^. Our analyses could not detect measurable amounts of other phytohormones, such as JA, JA-Isoleucine or the JA precursor 12-oxo-phytodienoic acid, and can suggest that jasmonate signalling is not induced in roots by *Trichoderma* colonisation. In agreement with our results, *Arabidopsis* and cucumber root interplay with *Trichoderma* involved increased expression of *Pal1* at 24 hpi and transient overexpression of *Lox*1^8,30,44^. JA synthesis and JA-mediated defences were suppressed also in oil palm roots at 3–12 weeks post inoculation with *T. harzianum*^31^. Et signalling appears also strongly suppressed at 24 hpi, as indicated by the downregulation of genes related to its synthesis (i.e. aminocyclopropane-1-carboxylic acid synthase, ACS and 1-aminocyclopropane-1-carboxylic acid oxidase, ACO) and response (i.e. Et-responsive factors, ERFs) (Fig. 6). Notably, sequence similarity with *A. thaliana* did not identify tomato DEGs related to *AtMYB72*, a root-specific TF that works upstream of JA and ET in ISR signalling induced in *Arabidopsis* by *Trichoderma*^8,46,47^. This result may be due to a different hormonal signalling occurring in tomato, and/or due to the recruitment of a so-far unidentified tomato transcriptional regulator lacking extensive sequence similarity with *AtMYB72*. Given the synergistic action between Et and auxin in the roots^48^, suppression of Et signalling was also suggested by the downregulation of key auxin biosynthetic (flavin monooxygenases, FMO-like) and responsive (e.g. auxin response factor, ARF) genes at 24 hpi.

As a result of the above signalling network, the plant immune response was overall transiently repressed at 24 hpi, as indicated by the downregulation of most genes within the enriched BP category 'response to stimulus' (Fig. 2b). Similarly, the MapMan bins ‘hormone metabolism’, ‘stress’ and ‘signalling’ showed overall downregulation at 24 hpi and upregulation at 72 hpi (Fig. 3). This response included the downregulation of Et/JA-responsive defence genes, such as defensins and terpene synthase (TPS) (Fig. 6) and complies with the strategy adopted by the plant to allow root colonisation by ISR-inducing microbes^6,8^. However, we observed also the upregulation of genes related to ROS detoxification (see above), defence (*PR1b1*; heat shock proteins, HSPs; and strictosidine synthase, STR) and cell wall strengthening (e.g. 4-coumarate:CoA ligase, 4CL; cinnamyl alcohol dehydrogenase, CAD; caffeoyl Co-A transferase, CCoAMT) (Fig. 6). These activated defences are mainly SA dependent and may be recruited to limit fungal spread within the roots, as was reported for the SA-impaired *sid2 A. thaliana* mutant interacting with *T. harzianum*^32,33^.

### Tomato root modulates its transcriptional machinery to further promote a positive interaction with *Trichoderma*

At later times of interaction (48–72 hpi), the observed root transcriptional response may be related to the known beneficial effects of *Trichoderma* in terms of plant protection (ISR priming and abiotic stress protection) and growth promotion^7,9,49^. In particular, the very low number of detected DEGs (80) seems to indicate 48 hpi as a transition point, in which the plant reprograms its transcriptional machinery mainly towards oxido-reduction (GSTs) and defence (HSPs, TPS) processes (Fig. 6). At 72 hpi, changes in SA-induced defences (e.g. dehydroascorbate reductase 2, *DHAR2*; GSTs; HSPs; Mildew Locus O, *MLO*; universal stress protein, USP) may contribute to hinder further *Trichoderma* penetration, but also possibly indicate induced priming. Consistent with our findings, expression of defence and ROS detoxification-related genes was systemically activated in several plant species during early interaction with *Trichoderma* spp.^11,13,14,35^ and was also stimulated during long-term interaction, taking part in the primed response to pathogens^4,14,31^.

Et and auxin signalling and altered redox balance can affect root architecture and stimulate root hairs^50^. This is also suggested by modulation of *SlMYB93*, similar to an auxin-induced negative regulator of lateral root development (*AtMYB93*)^51^. Modified root system together with ameliorated nutrient status suggested by modulation of nutrient transporters genes (Fig. 6) can improve plant growth. *Trichoderma*-induced hormone signalling and ROS scavenging may further contribute to plant growth through alleviation of environmental constraints^13,35^. Moreover, the observed increased expression of several HSP chaperone genes may be indicative of active protein synthesis, as previously suggested^12,14,52^.

### Perturbation of epiregulators expression, global DNA methylation and selective alterations of AS as emerging mechanisms modulating the *Trichoderma*–root mutualistic interaction

Recent studies have extended our understanding of the plant epigenetic control of pathogenesis and symbiosis^21,53,54^. Histone modifications and DNA methylation have emerged as critical regulators of defence priming, since they affect the transcription of defence-related genes through evolutionarily highly conserved functions^55^. Here we identified eight epigenetic modifiers as differentially expressed in treated roots, such as HAGs, AGO and RDM1. Moreover, we found an initial hypomethylation at 24 hpi followed by recovery and hypermethylation at 48 and 72 hpi, respectively. Consistently, independent studies found not only DNA demethylation at early stages of interaction with symbiotic microorganisms^56^, but also interplay between different histone modifications, which has been suggested to prime a part of SAR defence genes via mechanisms that are still poorly understood^53,54,57,58^. Overall, based on our data, it is tempting to speculate that the observed epiregulators expression changes mediate plant protection mechanisms primed by the interaction with *Trichoderma* and/or other *Trichoderma-*induced effects. Further studies with high-throughput capabilities for detecting unknown epigenetic changes are necessary to corroborate this hypothesis.

In addition, selective alterations of AS indicate that the *Trichoderma*–plant interplay is regulated also at the post-transcriptional level. In particular, alongside a general enrichment of the 'response to stimulus' BP term in both control and *Trichoderma*-treated roots, confirming that defence genes are frequently affected by AS^59^, the plant response to *Trichoderma* recruited at 24 hpi appears to involve specific AS events affecting defence gene *loci* (e.g. CC-NBS-LRRs, proteinase inhibitors I, major latex-like proteins). This finding evokes previous studies on plant–pathogen interactions reporting that most expressed genes are affected by AS, including plant defence genes^20,25,60^. Moreover, AS controls also root response to *Trichoderma* through modifications of transcriptional regulation, lipid and secondary metabolism.

To the best of our knowledge, our study is the first suggesting an involvement of epigenetic and post-transcriptional regulatory mechanisms during plant–*Trichoderma* interaction. However, further investigation is required to understand the dynamic engagement of these mechanisms during host–PGPF cross-talk.

**Fig. 8:**
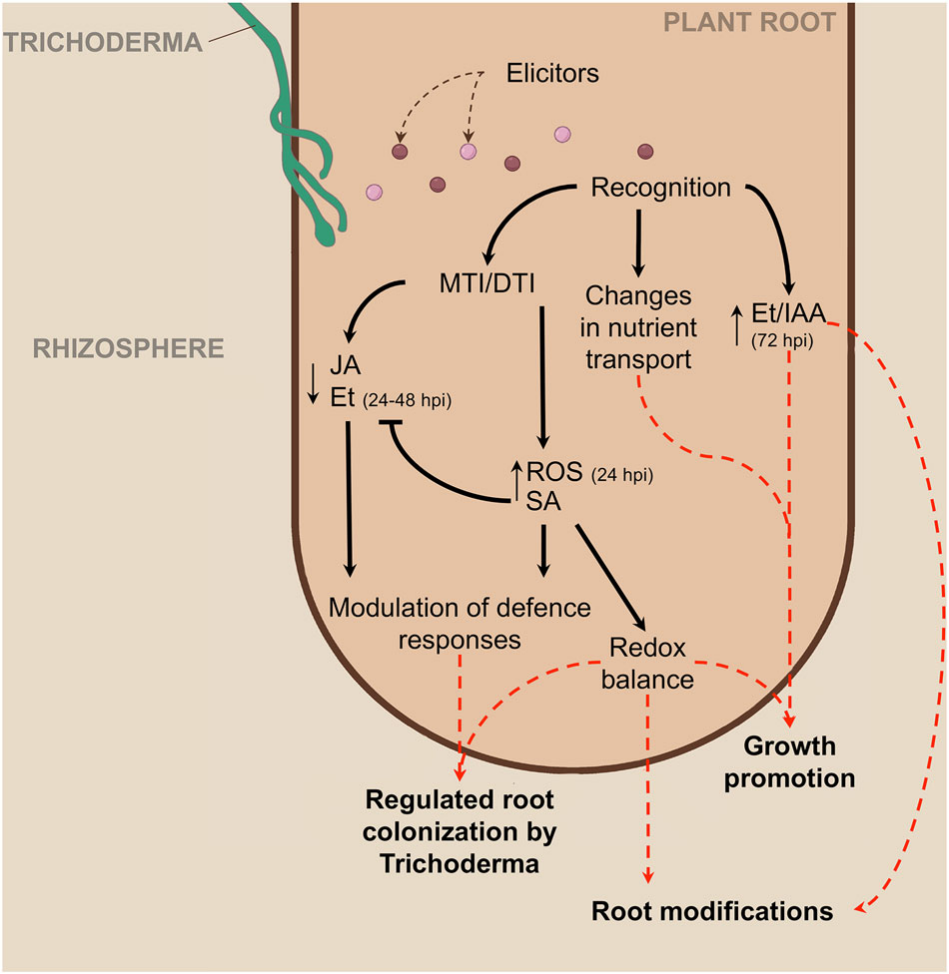
Proposed model of the early events occurring in the root–*Trichoderma* interaction. Recognition of *T. harzianum* MAMPs/DAMPs elicitors by tomato roots' pattern recognition receptors triggers MTI/DTI response across the observed interaction period (from 24 to 72 hpi). Phytohormone cross-talk orchestrates root colonisation by *Trichoderma*: (1) Induction of SA biosynthesis and signalling as well as ROS accumulation activate plant defence, thus limiting fungal spread and (2) SA-induced inhibition of JA and Et biosynthesis and signalling allows controlled root colonisation. At later times, increased Et and auxin signalling induce modifications of root architecture that, together with changes in nutrient transport, stimulate plant growth

Based on our results, we propose a putative model of the early tomato root–*T. harzianum* T22 interplay at the transcriptome level (Fig. 8). Plant–*Trichoderma* recognition appears to occur primarily through the MAMPs/DAMPs–plant membrane receptors system, inducing MTI responses. The main hormonal signalling is SA, mediating the host defence response to prevent spreading of fungal hyphae in the root vascular system. However, the root trascriptome reprogramming induced by the PGPF also includes suppression of both JA and Et biosynthesis and signalling, thus allowing root colonisation. At 72 hpi, increased transcription of Et and auxin signalling genes may result in alterations of root architecture (e.g. stimulation of root hairs). This process, together with modifications in nutrient transport occurring across the experimental period, is probably involved in the *Trichoderma*-induced plant growth stimulation. Our transcriptome analysis revealed also the activation of genes involved in ROS scavenging and plant defence throughout the observed interaction period. Both mechanisms may contribute to explain the known ability of *T. harzianum* to prime plant defences and alleviate the effects of abiotic stresses, although further investigation would be needed to validate this hypothesis.

This study of the interaction between tomato and *T. harzianum* contributes to a better understanding of the belowground events occurring between plants and beneficial rhizosphere fungi, though validation under natural conditions would be needed to account for the much higher environmental complexity. Our results may help to develop new strategies for crop biofertilization and bioprotection from biotic and abiotic stresses as well as to design new breeding strategies for the selection of crop varieties with improved ability to benefit from the interaction with beneficial rhizosphere fungi.

## Materials and methods

### Plant and fungal material

Tomato (*S. lycopersicum* cv. 'Crovarese') seeds and *T. harzianum* strain T22 were kindly provided by La Semiorto Sementi s.r.l. (Lavorate di Sarno, Italy) and the Department of Agricultural Sciences of the University of Naples Federico II (Portici, Italy), respectively. Seeds, sterilised in 2% sodium hypochlorite for 20 min and washed in sterile distilled water, were germinated in Petri dishes containing sterile filter paper in the dark at 25 °C. Seven-day-old spores of *T. harzianum* T22 cultured on potato dextrose agar (Sigma-Aldrich, St. Louis, Mo, USA) at 25 °C in the dark were collected by washing the plates with sterile distilled water and brought to a concentration of 10^8^ mL^−1^.

### Hydroponic growth conditions

Four-day-old tomato seedlings were transferred into hydroponic floating system. The plant nutrient solution (Colla G., personal communication) consisted, per litre, of 0.382 g Mg(NO_3_)_2_·6H_2_O, 0.812 g Ca(NO_3_)_2_·4H_2_O, 0.101 g KNO_3_, 0.320 g K_2_SO_4_, 0.205 g KH_2_PO_4_ and 0.014 g Hidromix S (Valagro, Atessa (CH), Italy), pH 6.4. Plants were grown in a walk-in growth chamber at 24/21 °C (day/night), 80% relative humidity and 16/8 h light/dark photoperiod. When tomato seedlings were 18 days old, the *T. harzianum* T22 inoculum was added to the nutrient solution to a final concentration of 10^5^ pre-germinated spores mL^−1^. Control plants were maintained in the nutrient solution without *Trichoderma*. Three biological replicates, each made by pooled roots from four randomly chosen plants, were harvested for the control and *Trichoderma*-inoculated plants (referred to as C and T along with the paper) at 24, 48 and 72 h post inoculation (hpi). Root samples were immediately frozen in liquid nitrogen and stored at −80 °C until RNA isolation.

### RNA isolation, cDNA library preparation and sequencing

Total RNA was extracted from washed tomato roots (100 mg) using the RNeasy Plant Mini Kit (Qiagen, Hilden, Germany), according to the manufacturer’s instructions. Complete DNA removal was obtained through on-column DNase I treatment using the RNase-Free DNase Set (Qiagen, Hilden, Germany). The quantity and purity of RNA was assessed using a NanoDrop ND-1000 spectrophotometer (Thermo Scientific, Wilmington, DE, USA). RNA integrity was further checked on a 2100 Bioanalyzer platform (Agilent Technologies, Santa Clara, CA, USA). Three biological replicates for each sample were used for RNA-seq. Libraries preparations and Illumina HiSeq 1500 sequencing service were carried out by Genomix4life S.r.l. (Baronissi, Italy, http://www.genomix4life.com). Raw reads (FASTQ format, 100 bp single end, Phred + 33) were quality filtered through the FASTX-Toolkit^61^, and sequencing adaptors were removed through the Trimmomatic software version 0.32^62^. High-quality reads were aligned onto the tomato reference genome (SL2.50) using TopHat2 version 2.0.11^63^. Only uniquely mapped reads were considered for downstream analysis. Reads that did not map onto the tomato genome were further aligned against the *T. harzianum* genome (v 1.0; http://genome.jgi.doe.gov/Triha1/Triha1.download.html). The HTSeq-count tool (HTSeq-0.6.1)^64^ was used to count the reads associated with each tomato or fungal gene.

### Differential gene expression analysis and gene ontology (GO) enrichment

Raw read counts were subjected to inter-sample normalisation by applying the Trimmed Mean of M-values method implemented in the EdgeR Bioconductor package (version 3.8.5)^65^. Differentially expressed genes (DEGs) were identified comparing results from both EdgeR and DESeq packages (version 1.18.0)^66^, setting a false discovery rate (FDR) of 10% (*P* < 0.1) and a minimum log_2_ fold change (FC) of ±1.1. Only DEGs called by both methods were used in downstream analysis. Differential gene expression in *T. harzianum* was estimated between the time points (24 vs. 48 hpi, 48 vs. 72 hpi and 24 vs. 72 hpi). In this case, the EdgeR tool was used for DEG call; quality parameters (FDR and minimum FC) were fixed as previously described. Enrichment analysis of GO terms, included in the MF and BPs domains, was performed by the topGO package, version 2.18.0^67^. Identified DEGs were mapped to MapMan bins for data visualisation and pathway analysis (version 3.6.0)^68^. To this end, the tomato MapMan ontologies (http://www.gomapman.org/export/current/mapman/sly_SL2.40_ITAG2.3_2015-01-09_mapping.txt.tgz) were retrieved from the GOMapMan web resource^69^ and imported in the MapMan tool. The Multiple Experiment Viewer v.4.9.0 was used for clustering analysis and heatmap generation^70^. Self-organising tree algorithm (SOTA) was used to cluster genes on the basis of their expression profiles.

### RNA-seq validation by quantitative RT-PCR

RT-qPCRs were performed on 12 randomly selected genes to validate the RNA-seq data. Primer design was carried out using the PrimerQuest tool (http://eu.idtdna.com/Primerquest/Home/Index). Primer sequences are listed in Table S1. The same RNA samples used for sequencing were also employed for PCR-based expression analysis. DNase-treated total RNA (1 μg) was reverse transcribed using SuperScript II Reverse Transcriptase (Life Technologies, Carlsbad, CA, USA) following the protocol previously described in Tucci et al.^4^. Each PCR reaction consisted of 5 µL of 1:20 diluted cDNA, 6.25 µL of 2X SYBR^®^ Select Master Mix (Life Technologies, Carlsbad, CA, USA) and 0.2 µM of each gene-specific primer in a total volume of 12.5 μL. qRT-PCRs were performed using a 7900HT fast RT-PCR System (Applied Biosystems, Foster City, CA, USA). PCR cycling conditions were 10 min at 95 °C (1 cycle), followed by 40 cycles of two steps at 95 °C for 15 s and 60 °C for 1 min. Melting curves (60–95 °C) were recorded at the end of each run in order to check the specificity of the amplification products. Three biological replicates with three technical repetitions were tested. The ΔΔCt method was used for relative gene expression analysis^71^. The elongation factor *EF1-α* gene (GenBank Acc. No. NM_001247106.2) was used as an endogenous reference gene to normalise the gene expression values. The relative expression of each gene was calculated by using the untreated control at the same time point as a calibrator. PCR efficiencies calculated by the standard curve method were 87–107% for each primer pairs with a correlation coefficient (*R*^2^) of 0.99.

### Quantification of *T. harzianum* tomato root colonisation by qRT-PCR

The presence of *Trichoderma* was quantified in the control and T22-treated plants at 24, 48 and 72 hpi. First-strand cDNA synthesis from root RNA and qRT-PCRs were conducted using the conditions described above, except for cDNA that was diluted 1:40 for the tomato EF1-α gene and 1:10 for the *T. harzianum* beta actin gene^72^, respectively. The EF1-α gene was used as an endogenous reference gene to normalise the gene expression values. The relative expression of *T. harzianum* actin was calculated by using the untreated control at 24 hpi as a calibrator.

### Determination of global DNA methylation

Total DNA was isolated from the same plant materials used for RNA-seq analysis. The DNeasy Plant Mini Kit (Qiagen, Dusseldorf, Germany) was used following the manufacturer’s protocol. Quantity and quality of the isolated DNA were measured using the NanoDrop ND-1000 spectrophotometer (Thermo Scientific, Wilmington, DE, USA) and Qubit 2.0 fluorometer (Life Technologies, Carlsbad, CA). For each sample, the absolute level of global DNA methylation was assessed in percentages of 5-methylCytosine (% 5-mC) using the MethylFlash Methylated DNA Quantification Kit (Epigentek, New York, NY). The amount of methylated DNA (proportional to the OD intensity measured at 450 nm) was measured using the microplate reader spectrophotometer Multiskan FC (Thermo Scientific, Wilmington, DE, USA). % 5-mC (methylated polynucleotide containing 50% of 5-methylCytosine) was calculated using the formula described in the kit manual. Biological replicates were analysed in triplicate. The *t*-test was applied to discriminate amongst data averages at 95% confidence level (*p* ≤ 0.05) using the Visual Basic for Applications macro programme of the Microsoft Office Excel 2007 package.

### Analysis of alternative transcripts

R-SAP (RNA-Seq Analysis Pipeline)^73^ was used to compare the read-to-reference genome alignments with all tomato gene tracks, as annotated in the file *ITAG2.4_gene_models.gff3* downloaded from the SGN ftp server (ftp://ftp.solgenomics.net/genomes/Solanum_lycopersicum/annotation/ITAG2.4_release/) in order to detect the novel transcribed isoforms and/or identify novel gene features. We firstly converted the tomato annotation gene tracks from the GFF3 to the GTF format using the *gffread* programme that comes with Cufflinks^74^. Then, we convert each SAM/BAM file into the PSLX format. We performed multiple R-SAP runs (one *per* Illumina read dataset) with IdentityCutoff = 95 and CovCutoff = 98. Finally all the results were collapsed into a unique data report for downstream analysis. AgriGO v2.0^75^ was used to perform singular enrichment analysis (SEA) on genes affected by AS and specific for control and treated samples. REVIGO^76^, with the parameter 'allowed similarity' set to 'small(0.5)', was used to reduce redundancy and produce a summary of the enriched GO terms.

### Detection and quantification of plant hormones

Analysis and quantification of SA, SAG, jasmonic acid (JA) and JA-Ile conjugate was carried out by high liquid chromatography–tandem mass spectrometry (HPLC–MS/MS) analysis, according to Schenk et al.^77^ with some modifications. Finely ground root material was extracted twice with 1 mL of methanol by ultrasound-assisted solid–liquid extraction (UA-SLE) for 30 min and centrifuged at 14,000 rpm for 2 min. The supernatants were collected and evaporated in a speed-vac at 30 °C and redissolved in 250 μL methanol/water 4:6, v/v. Quantitative HPLC–MS/MS analysis was performed using a Shimadzu Nexera X2 UHPLC system (Shimadzu, Milano, Italy) coupled to an Q-TRAP-6500 mass spectrometer (AB Sciex, Milan, Italy) operating in negative ion mode. The extracts were separated on a Kinetex C18 column (100 × 2.1 mm, 2.7 µm; Phenomenex, Bologna, Italy) using a gradient elution of H_2_O (A) and acetonitrile (B), both containing formic acid 0.05%, v/v. After injection (10 µL), the analytes were eluted using the following gradient: 0–5 min, 2% B, 5–12 min, 2–25% B, 12–16 min, 25–98% B. The column was kept at 30 °C and the flow rate was set at 0.4 mL min^−1^ for all chromatographic runs. At the end of each run, the column was washed with 98% B (5 min) and re-equilibrated with 2% B (5 min). In order to improve the analyte ionisation and to select the multiple reaction monitoring (MRM) transition, tune optimisation was carried out by the direct infusion of SA standard solution at a concentration of 2 µg mL^−1^. The optimal instrument parameters were as follow: ion spray voltage (IS) −4500 V, source temperature (TEM) 400 °C, dwell time was 20 ms for each MRM transition, nebulizer gas (GS1) 40 psi, heater gas (GS2) 40 psi, curtain gas (CUR) 35 psi and declustering potential [−110 VL]. For the quantification and identification of analytes, MRM was used, monitoring the precursor/product transitions: *m*/*z* 136.9 → 93.0 (collision energy [CE] −20 V) and 136.9 → 65.0 (CE −36 V) for SA; *m*/*z* 209.1 → 59.0 (CE −25 V) for JA; *m*/*z* 290.1 → 165.1 (CE −24 V) for 12-oxo-phytodienoic acid; *m*/*z* 322.2 → 130.1 (CE −30 V) for JA-Ile conjugate and *m*/*z* 299.0 → 136.9 (CE −25 V) for SAG. Both Q1 and Q3 quadrupoles were maintained at unit resolution. Analyst™ software version 1.6 was used for mass spectrometer control and data acquisition/processing.

Calibration external standard method was used to quantify SA in root extracts using seven SA concentration levels (1–62.5 ng mL^–1^, triplicate injections for each level). The regression curve was tested with the analysis of variance (ANOVA), and a linear model was found appropriate over the tested concentration range (*y* = 4276.4*x* + 43829; *R*^2^ = 0.99). The SA amount was finally expressed as ng per gram of fresh weight ± deviation standard (*n* = 3). SAG levels were expressed as SA equivalents.

### Data availability statements

The raw sequences from control tomato plants can be found in the European Nucleotide Archive under the study accession number PRJEB20101 (https://www.ebi.ac.uk/ena/data/view/PRJEB20101) from sample accession number ERS1623272–ERS1623280.

The raw sequences from *Trichoderma*-treated tomato plants can be found in the European Nucleotide Archive under the study accession number PRJEB21256 (https://www.ebi.ac.uk/ena/data/view/PRJEB21256).

## Electronic supplementary material

Supporting information Figures S1_S6

Table S1

Table S2

Table S3

Table S4

Table S5
