# Supplementary material for: Transcriptome reprogramming, epigenetic modifications and alternative splicing orchestrate the tomato root response to the beneficial fungus Trichoderma harzianum
Source: Hortic Res. 2019 Jan 1;6:5. doi: 10.1038/s41438-018-0079-1 (PMC6312540; doi:10.1038/s41438-018-0079-1)
Supplement: Supplementary file 1 — Supporting information Figures S1_S6 [file 41438_2018_79_MOESM1_ESM.docx]

***Supplementary material***

**Transcriptome reprogramming, epigenetic modifications and alternative splicing orchestrate the tomato root response to the beneficial fungus *Trichoderma harzianum***

Monica De Palma, Maria Salzano, Clizia Villano, Riccardo Aversano, Matteo Lorito, Michelina Ruocco, Teresa Docimo, Anna Lisa Piccinelli, Nunzio D’Agostino*, Marina Tucci*

*Corresponding authors

Marina Tucci e-mail: mtucci@unina.it

Nunzio D’Agostino e-mail: nunzio.dagostino@crea.gov.it

**
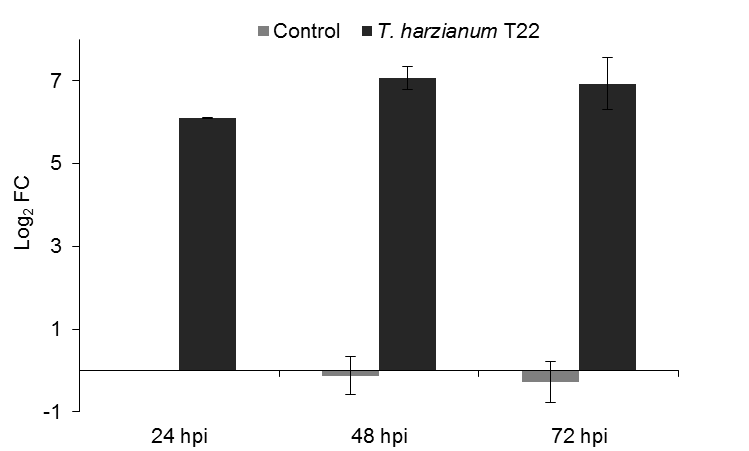
**

**Supplementary Figure S1.** Quantiﬁcation of *T. harzianum* T22 in tomato roots by qRT-PCR. *T. harzianum* beta actin gene expression levels for each time point are reported as means of the fold increase relative to that of the untreated control at 24 hpi. The tomato Elongation factor (EF1-α) gene was used as an internal control to normalize the expression level. Bars represent standard deviations of the means of three biological replicates.

**A)**

**
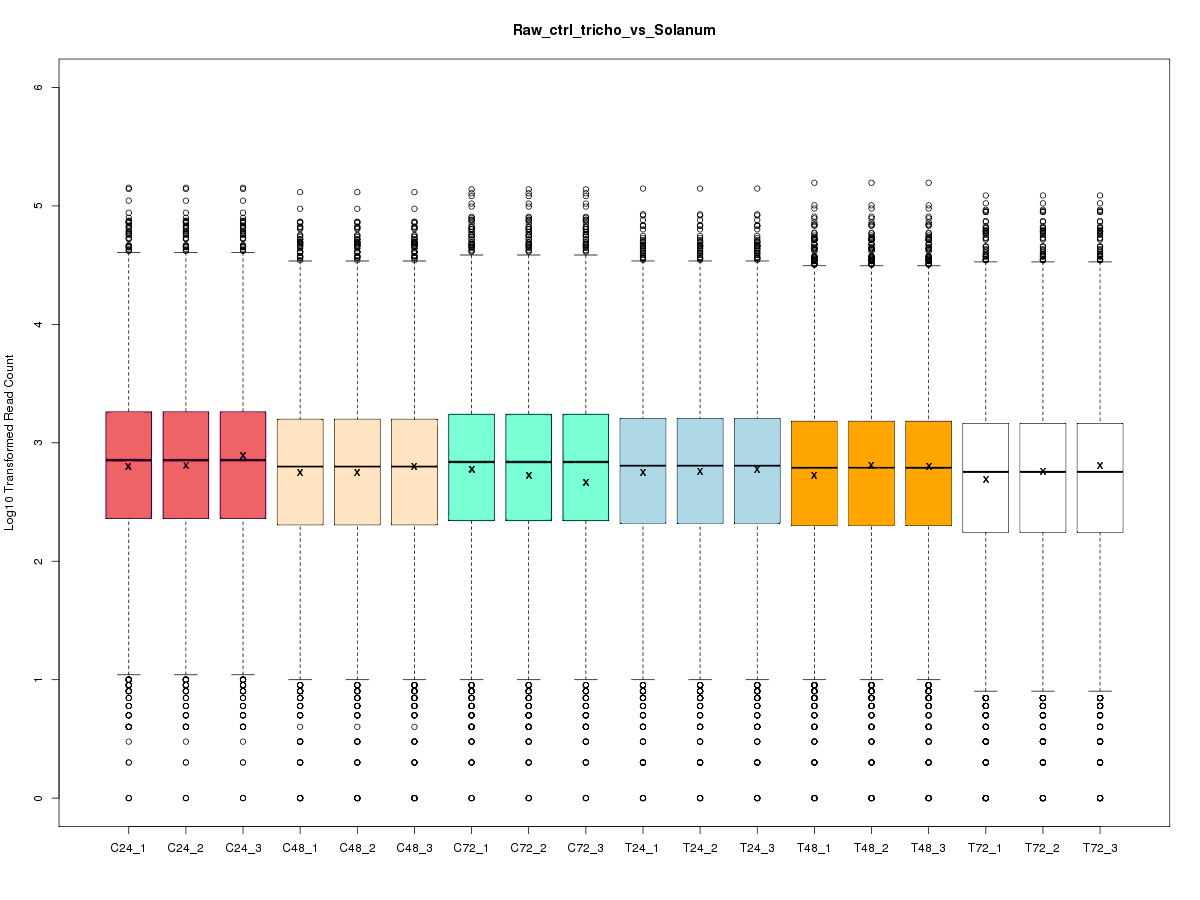
**

**B)
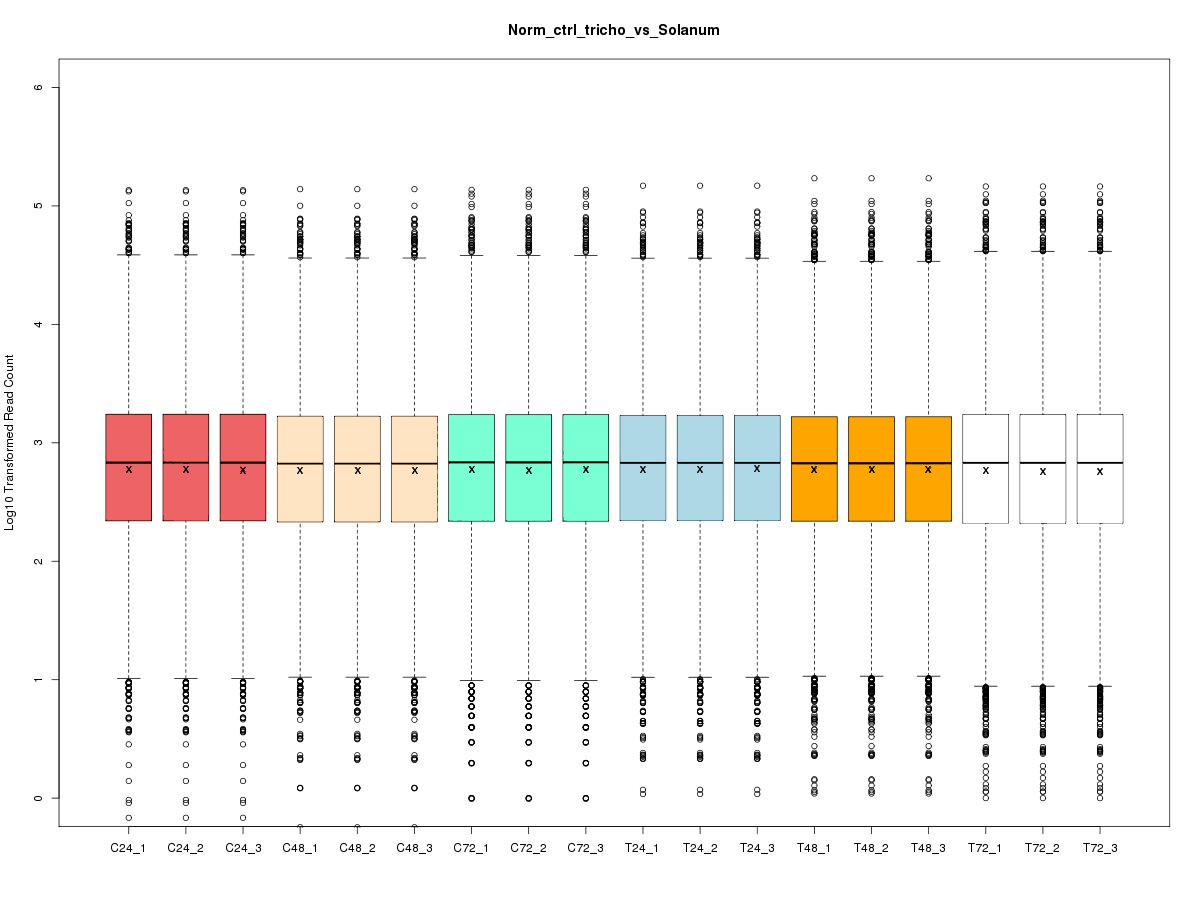
**

**Supplementary Figure S2.** Box plots of the distribution of read counts before (A) and after TMM normalization (B). Biological replicates of the 6 experimental points are represented with the same colour.

**A)**


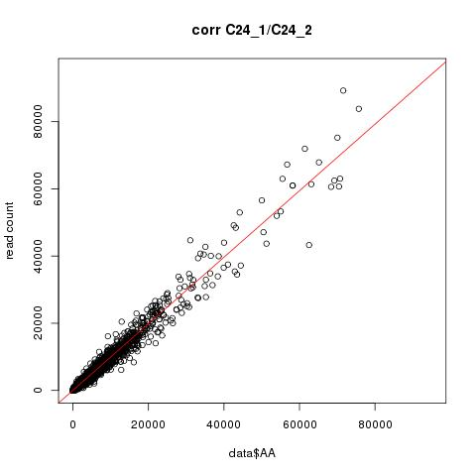

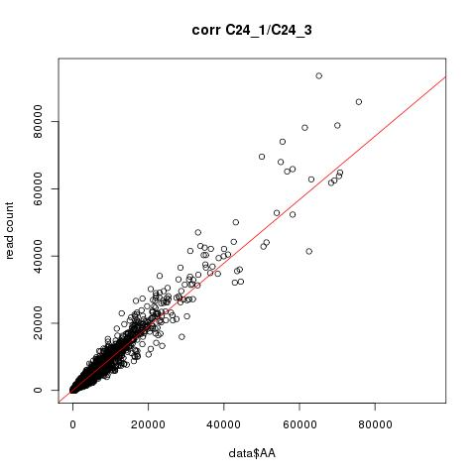

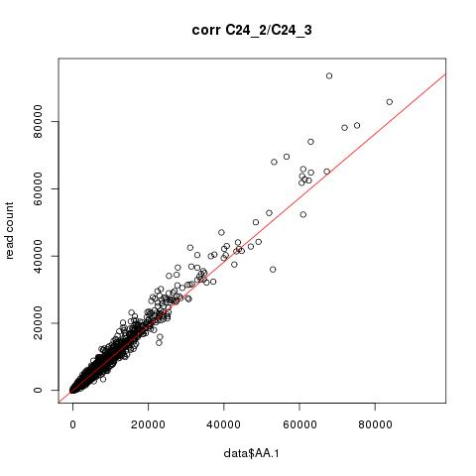


**r^2^ = 0.987**


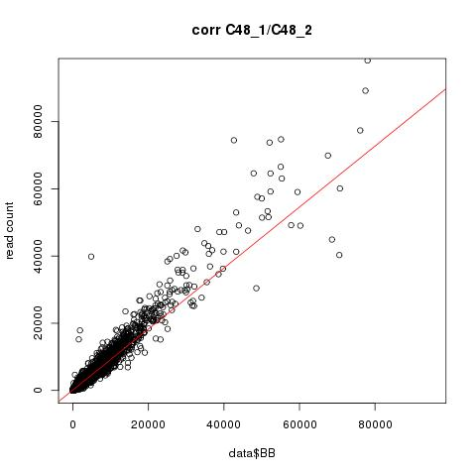

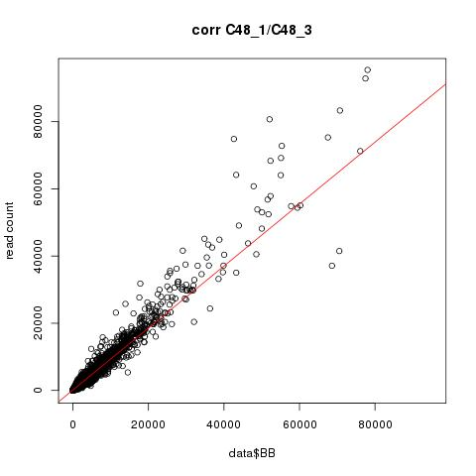

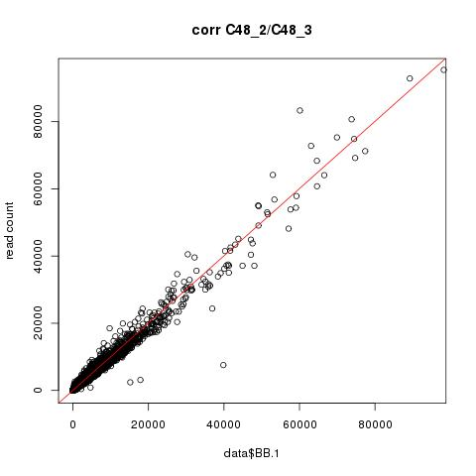


**r^2^ = 0.982**


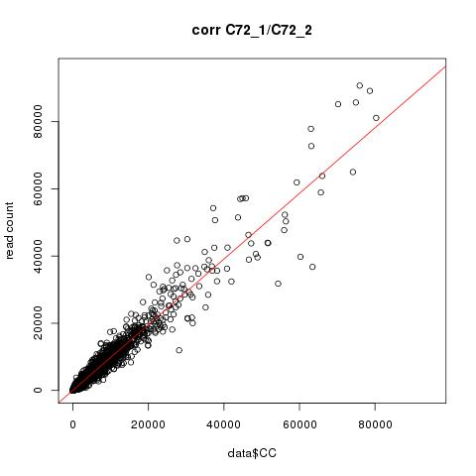

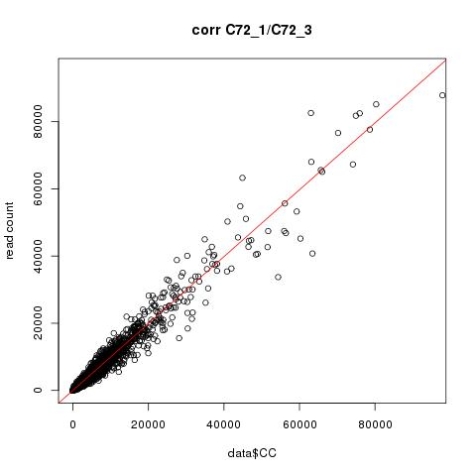

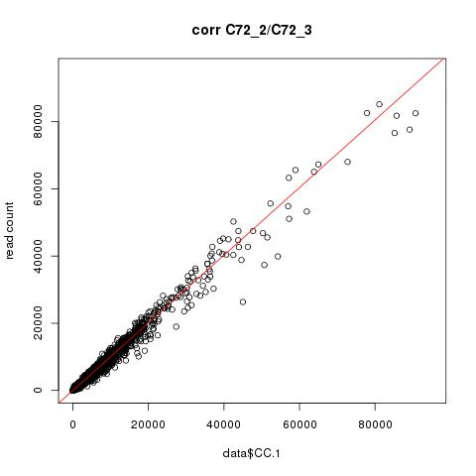


**r^2^ = 0.986**

**B)**


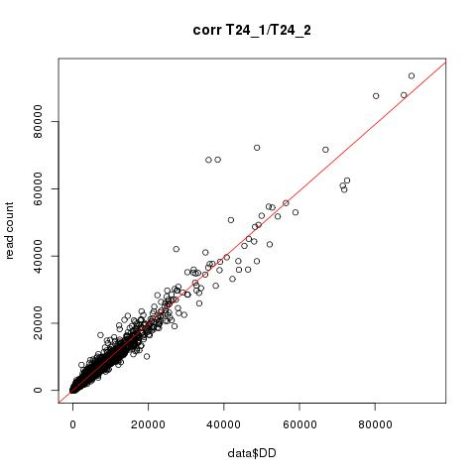

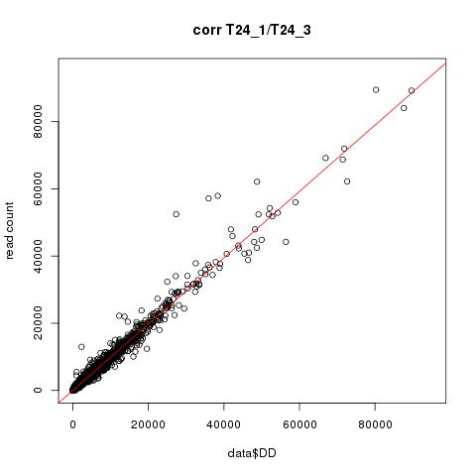

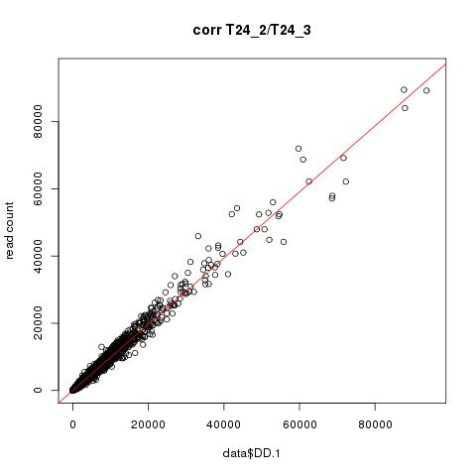


**r^2^ = 0.989**


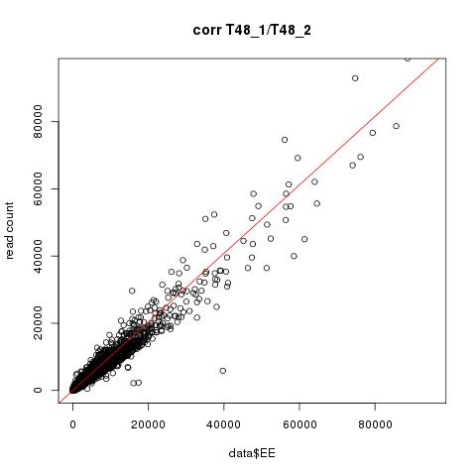

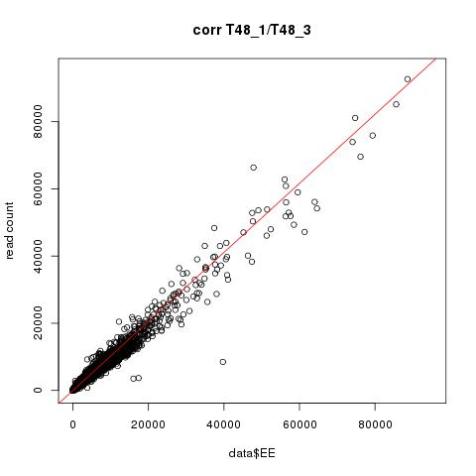

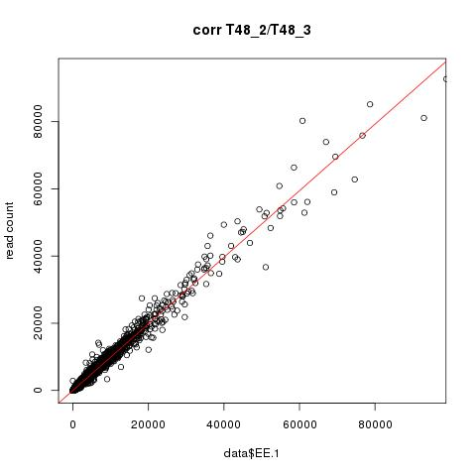


**r^2^ = 0.985**


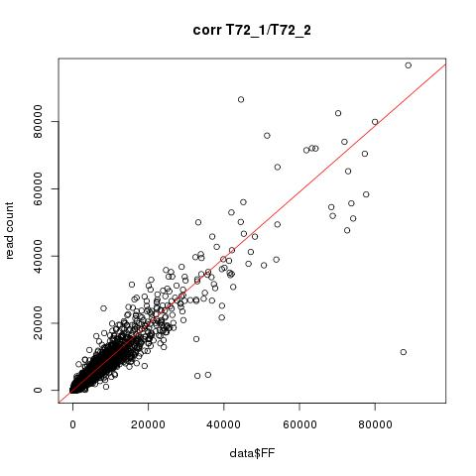

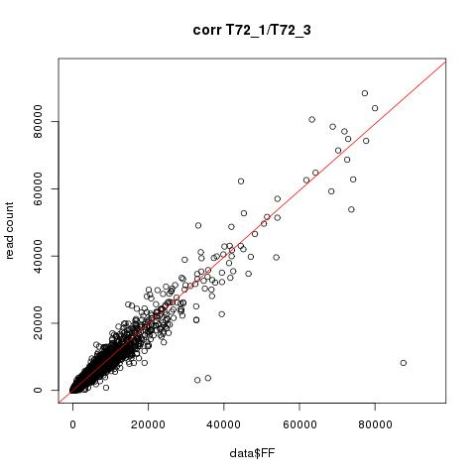

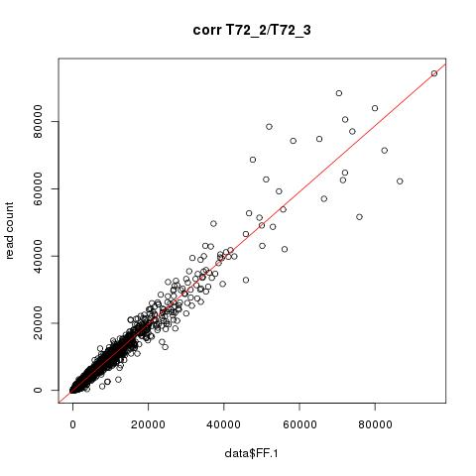


**r^2^ = 0.975**

**Supplementary Figure S3.** Scatter plots showing the relationships between the expression estimates in three biological replicates of A, control (C) and B, *T. harzianum* T22-treated (T) samples at the three experimental time points. Average Pearson’s correlation coefficients (r^2^) are based on the read counts of all tomato genes of each biological replicate.

24 hpi 48 hpi 72 hpi

**Supplementary Figure S4**. Comparison between RNA-seq and RT-qPCR expression values of 12 randomly selected DEGs. Linear regression between RNA-seq (x axis) and real time RT-PCR (y axis) fold increase (Log_2_FC) in gene expression levels relative to that of untreated control is reported for the three experimental time points (24, 48 and 72 hpi). The tomato elongation factor gene was used as an internal normalization control. *SAMT*, Salicylic acid carboxyl methyltransferase; *AGO4*, Argonaute 4-like protein; *RdM1*, RNA-directed DNA methylation 1; *PR1b1*, Pathogenesis-related protein 1b; *HSP22*, Heat shock protein 22.


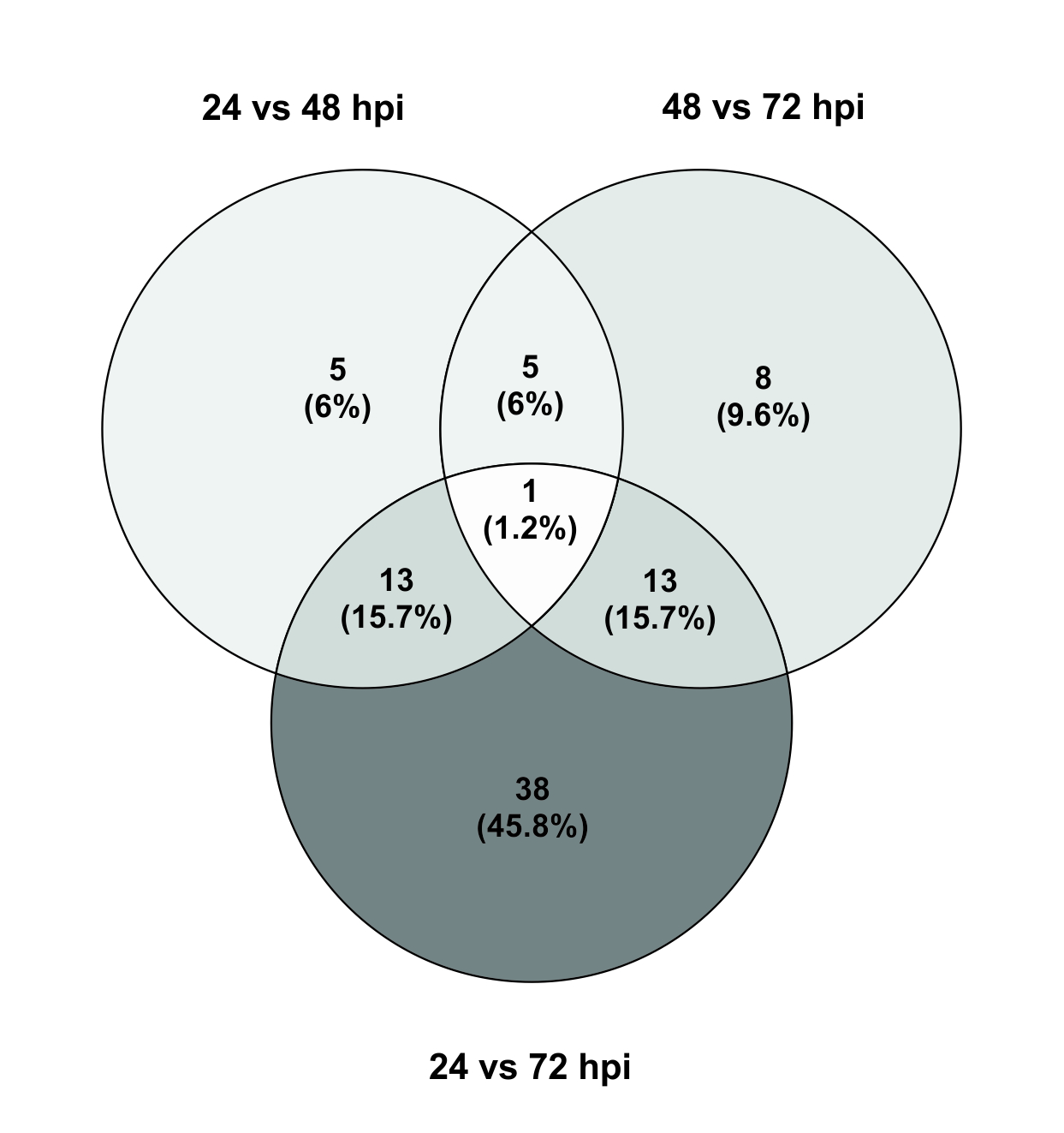


**Supplementary Figure S5**. Venn diagram describing the distribution of differentially expressed *T. harzianum* T22 genes after different times of interaction. False Discovery Rate was set at 10% (P < 0.1) and minimum fold change at ±1.1.


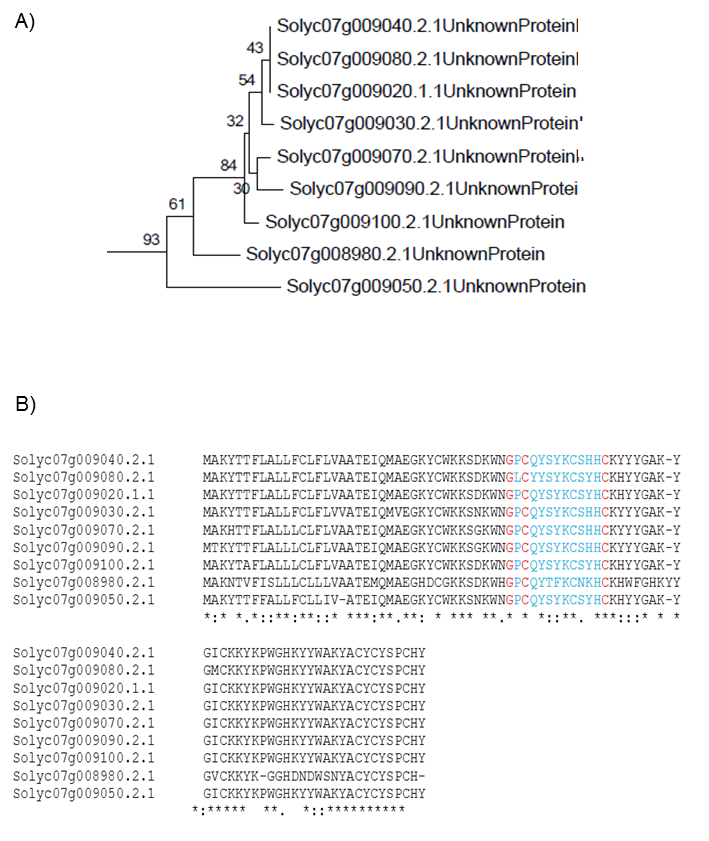


**Supplementary Figure S6.** Clustering (A) and alignment (B) of the amino acid sequences of nine proteins annotated as unknown in the MapMan bin 35. The amino acids of the gamma-core motif (GXCX3-9C), common to defensin proteins, are indicated in colour. The neighbour-joining phylogenetic tree was produced using the MEGA6 tool.
